# Unilateral Microelectrode Recording–Guided Pallidotomy in Advanced Parkinson's Disease: Clinical and Neuropsychological Outcomes in a Guatemalan Cohort

**DOI:** 10.31083/RN44171

**Published:** 2026-02-09

**Authors:** Juan Carlos Lara-Girón, Diego Sánchez-Díaz, Abel Alejandro Sanabria-Sanchinel, Edwin Stanly Escobar-Pineda, Manuel Hernández-Salazar, Marco Díaz, Víctor Pineda, Williams González, Raúl Cardona, Zoe María Gómez

**Affiliations:** ^1^Neurosciences Department, Humana Center for Epilepsy and Functional Neurosurgery Center, 01010 Guatemala City, Guatemala; ^2^Department of Neurosurgery, “20 de Noviembre” National Medical Center, Institute for Social Security and Services for State Workers (ISSSTE), 03104 Mexico City, Mexico

**Keywords:** Parkinson's disease, pallidotomy, internal globus pallidus, stereotactic surgery, radiofrequency ablation, neuropsychology, microelectrode recording (MER), enfermedad de Parkinson, palidotomía, globo pálido interno, cirugía estereotáctica, ablación por radiofrecuencia, neuropsicología, registro con microelectrodos (MER)

## Abstract

**Background:**

Unilateral microelectrode-guided pallidotomy has re-emerged as a therapeutic option for advanced Parkinson's disease (PD), particularly in resource-limited settings. This study evaluated motor and neuropsychological outcomes following radiofrequency ablation of the internal globus pallidus (GPi) using image fusion and intraoperative microelectrode recording. To assess the motor efficacy, cognitive impact, and safety profile of unilateral GPi pallidotomy guided by neurophysiological monitoring in patients with advanced idiopathic PD of the rigid-akinetic subtype.

**Methods:**

This retrospective, single-center, observational case series included 12 patients with advanced PD who underwent unilateral radiofrequency pallidotomy targeting the internal segment of the GPi. Movement Disorder Society–Unified PD Rating Scale Part III (MDS-UPDRS-III), including both contralateral and ipsilateral domain sub scores. Levodopa-induced dyskinesias (UPDRS-IV) and cognitive performance (NEUROPSI Attention and Memory battery) were also assessed before and 12 months after surgery. Statistical analyses were conducted using paired *t*-tests or Wilcoxon signed-rank tests depending on data distribution, with significance set at *p* < 0.05.

**Results:**

At 12-month follow-up, motor outcomes improved significantly. Mean MDS-UPDRS-III scores decreased from 64.1 ± 27.1 preoperatively (OFF medication) to 37.8 ± 24.4 postoperatively, and from 23.5 ± 17.0 to 10.6 ± 8.5 in the ON state. The overall OFF-state improvement was 44.4% ± 21.2%. Paired *t*-tests confirmed a highly significant reduction in motor scores (*t* = 6.19, *p* < 0.0001; mean change –26.3 points, 95% confidence intervals (CI) –34.7 to –18.0). Domain-specific analysis showed significant contralateral improvements in rigidity (–48%, *p* = 0.0006), bradykinesia (–49.5%, *p* < 0.0001), resting tremor (–81%, *p* = 0.004), gait (–27.8%, *p* = 0.013), postural stability (–39%, *p* = 0.021), and dyskinesias (–56%, *p* = 0.017). Ipsilateral changes were mild and not statistically significant for rigidity (–14.6%, *p* = 0.519), bradykinesia (–25.0%, *p* = 0.084), or rest tremor (–50.0%, *p* = 0.121). No major surgical complications occurred. Neuropsychological assessment revealed modest postoperative gains in executive functioning and working memory, with preservation of global cognition.

**Conclusion:**

Unilateral GPi pallidotomy guided by imaging and microelectrode recording is a safe and effective procedure that significantly improves motor symptoms while preserving cognitive function in appropriately selected patients with advanced PD, as demonstrated in our Guatemalan cohort.

## Abstract (es)

**Antecedentes:**

La palidotomía unilateral guiada por microelectrodo ha resurgido como una opción terapéutica para la enfermedad de Parkinson (EP) avanzada, particularmente en entornos con recursos limitados. Este estudio evaluó los resultados motores y neuropsicológicos tras la ablación por radiofrecuencia del globo pálido interno (GPi) utilizando fusión de imágenes y microrregistro intraoperatorio. Evaluar la eficacia motora, el impacto cognitivo y el perfil de seguridad de la palidotomía unilateral del GPi guiada por monitoreo neurofisiológico en pacientes con EP idiopática avanzada del subtipo rígido-acinético.

**Métodos:**

Se realizó una serie de casos retrospectiva, observacional y de un solo centro, que incluyó a 12 pacientes con EP avanzada sometidos a palidotomía unilateral por radiofrecuencia dirigida al segmento interno del GPi. La función motora se evaluó mediante la Escala Unificada de Evaluación de la EP, Parte III (MDS-UPDRS-III), incluyendo subpuntuaciones de dominios contralaterales e ipsilaterales. Las discinesias inducidas por levodopa (UPDRS-IV) y el rendimiento cognitivo (batería NEUROPSI de Atención y Memoria) se evaluaron antes de la cirugía y a los 12 meses de seguimiento. Los análisis estadísticos se realizaron utilizando pruebas t pareadas o pruebas de rangos con signo de Wilcoxon según la distribución de los datos, estableciendo la significancia estadística en *p* < 0,05.

**Resultados:**

A los 12 meses de seguimiento, los resultados motores mostraron una mejoría significativa. Las puntuaciones medias de la MDS-UPDRS-III disminuyeron de 64,1 ± 27,1 en el preoperatorio (estado OFF medicación) a 37,8 ± 24,4 en el postoperatorio, y de 23,5 ± 17,0 a 10,6 ± 8,5 en el estado ON. La mejoría global en estado OFF fue del 44,4% ± 21,2%. Las pruebas t pareadas confirmaron una reducción altamente significativa de las puntuaciones motoras (t = 6,19, *p* < 0,0001; cambio medio –26,3 puntos, intervalos de confianza (IC) del 95%: –34,7 a –18,0). El análisis por dominios mostró mejorías contralaterales significativas en rigidez (–48%, *p* = 0,0006), bradicinesia (–49.5%, *p* < 0,0001), temblor en reposo (–81%, *p* = 0,004), marcha (–27,8%, *p* = 0,013) y estabilidad postural (–39%, *p* = 0,021), así como en las discinesias (–56%, *p* = 0,017). Los cambios ipsilaterales fueron leves y no estadísticamente significativos para rigidez (–14,6%, *p* = 0,519), bradicinesia (–25,0%, *p* = 0,084) y temblor en reposo (–50,0%, *p* = 0,121). No se presentaron complicaciones quirúrgicas mayores. La evaluación neuropsicológica reveló modestos incrementos postoperatorios en funciones ejecutivas y memoria de trabajo, con preservación de la cognición global.

**Conclusión:**

La palidotomía unilateral del GPi guiada por imágenes y registro con microelectrodos es un procedimiento seguro y eficaz que mejora significativamente los síntomas motores, preservando la función cognitiva en pacientes adecuadamente seleccionados con EP avanzada, como se demuestra en esta cohorte guatemalteca.

## 1. Introduction

Parkinson’s disease (PD) is a progressive and irreversible neurodegenerative disorder, characterized by bradykinesia, rigidity, and tremor as its cardinal manifestations [1]. Prior to the introduction of levodopa, surgery was a widely used strategy for the treatment of movement disorders. In 1909, Horsley described the resection of the precentral gyrus to treat hemiathetosis [2]. In 1937, Busy and Case reported motor area resection for the treatment of tremor [2]. In 1939, Meyers performed the first direct intervention on the basal ganglia by resecting two-thirds of the caudate nucleus to treat postencephalitic tremor [2]. Subsequently, Cooper incidentally observed the disappearance of tremor after sectioning the anterior choroidal artery during a pedunculotomy; he later ligated the same artery in a series of 55 patients. However, due to the high complication rates, the procedure was eventually abandoned [3].

The most significant advancement in movement disorder surgery was the introduction of stereotactic apparatuses by Spiegel *et al*. [4]. Since then, various stereotactic systems have been developed based on different principles, all of which have employed pneumoencephalography and validated stereotactic atlases. Currently, stereotactic navigation planning software is available, allowing fusion of magnetic resonance imaging and computed tomography, achieving submillimetric accuracy.

In 1953, Narabayashi and Okuma [5] reported the effects of chemical pallidotomy in patients with PD. Guiot and Brion [6] reported electrocoagulation of the pallidum, making it a frequent surgical target, particularly in the anterodorsal region. However, long-term outcomes were limited, with rigidity control being the main sustained benefit. In 1992, Leksell redirected the coordinates toward the ventroposterolateral region of the internal globus pallidus (GPi), achieving improved and lasting results [7].

This series provides recent data from a Latin American center using microelectrode-guided pallidotomy, with contemporary stereotactic techniques, standardized motor and neuropsychological assessments, and one-year follow-up.

## 2. Materials and Methods

### 2.1 Study Design and Setting

This retrospective, single-center observational case series included twelve patients with advanced Parkinson’s disease (rigid–akinetic phenotype with dyskinesias) who completed the standardized preoperative protocol between January and December 2022. The preoperative protocol comprised high-resolution magnetic resonance imaging, multidisciplinary clinical evaluations, and comprehensive neuropsychological testing using the NEUROPSI Attention and Memory battery.

All cases were reviewed by the Movement Disorders Pre-Surgical Committee, which determined candidacy for unilateral pallidotomy based on disabling clinical features, primarily akinesia and rigidity with unilateral predominance. Patients with alternative clinical phenotypes were evaluated for either lesioning or deep brain stimulation in other targets, depending on their specific presentation.

The study was approved by the Institutional Ethics Committee (HUMANA-CEI-2024-003, 12 February 2024). Trial registration was not applicable.

### 2.2 Primary Outcome

Evaluations were conducted under standardized conditions in both the OFF- and ON-medication states. The OFF state was obtained after overnight withdrawal of dopaminergic therapy, with the last dose taken with the evening meal on the night before evaluation, resulting in a 16–18-hour medication washout in accordance with accepted clinical protocols. The ON state was assessed after administration of each patient’s usual dopaminergic regimen. For statistical analysis, paired differences were tested for normality using the Shapiro–Wilk test. As assumptions of normality were met, comparisons were performed with paired Student’s *t*-tests. Outcomes are presented as means with standard deviations, mean changes, 95% confidence intervals (CI), and *p*-values. Statistical significance was defined as *p* < 0.05.

### 2.3 Secondary Motor Outcomes

Secondary outcomes were derived from selected MDS-UPDRS Part III items, evaluated independently in the preoperative OFF state and at postoperative follow-up. Item selection was defined a priori, based on (1) the domains most reliably assessed in unilateral procedures, where contralateral hemibody performance is clinically most relevant, and (2) the use of items that provide direct, isolated measures of the motor features of interest, rather than mixed or multi-component tasks. As the MDS-UPDRS does not prescribe validated subscore groupings for rigidity, bradykinesia, or tremor, these operational definitions follow approaches commonly used in unilateral lesion and DBS surgery series.

•**Rigidity**: contralateral extremities (items 3.3B/3.3C for upper limb, 3.3D/3.3E for lower limb) and ipsilateral extremities using the same items.

•**Bradykinesia**: contralateral finger tapping (item 3.4A/3.4B) and toe tapping (item 3.7A/3.7B); ipsilateral bradykinesia was calculated using the same items, expressed as the average score of finger and toe tapping (0–4 scale).

•**Resting tremor**: contralateral extremities (items 3.17A/3.17B for upper limb, 3.17C/3.17D for lower limb) and ipsilateral extremities using the same items.

•**Gait**: item 3.10.

•**Postural stability**: item 3.12.

•**Dyskinesias**: Part IV, item 4.1 (time spent with dyskinesias in the ON-medication state).

All ratings were performed by the same neurologist with formal training in MDS-UPDRS administration. As with the primary outcome, paired differences were tested for normality using the Shapiro–Wilk test. Normally distributed variables were compared using paired Student’s *t*-tests, while non-parametric variables were analyzed with the Wilcoxon signed-rank test. Results are reported as mean changes with 95% CIs, percentage improvements, and *p*-values, with statistical significance defined as *p* < 0.05.

### 2.4 Neuropsychological Outcomes

Neuropsychological performance was evaluated using the NEUROPSI battery, which comprises 18 domains assessing orientation, attention, memory, language, executive functioning, visuospatial skills, and motor control. This instrument was selected because it has been standardized and normed in Hispanic and Latin American populations, providing culturally appropriate assessment of cognitive functions. All patients completed the full protocol pre- and postoperatively. Scores range from 0 to 19 points per domain, with higher scores reflecting better performance. Comparisons were performed using paired tests (Student’s *t* or Wilcoxon, as appropriate), and effect sizes were calculated for within-subject differences.

### 2.5 Surgical Planning and Procedure

Prior to the intervention, patients were provided with detailed information about the procedure, including its objectives, potential benefits, risks, and therapeutic alternatives. After ensuring full understanding and addressing all questions, informed consent was obtained explicitly and in writing.

Subsequently, under local anesthesia with scalp block, a Cosman-Roberts-Wells (CRW) stereotactic frame was placed for surgery. A computed tomography (CT) scan was performed using a SOMATOM X.ceed scanner (Siemens Healthineers, Erlangen, Germany) and fused with magnetic resonance imaging (MRI) obtained using a 1.5-Tesla scanner (Siemens Healthineers) using the WayPoint™ Navigator software (version 4.6.6; FHC Inc., Bowdoin, ME, USA). The stereotactic targets within the GPi were identified indirectly using x, y, and z coordinates referenced to the intercommissural line, with support from the Morel atlas. The target and stereotactic trajectory were refined, with final validation performed directly by visualizing the GPi on T2-weighted MRI sequences in all planes.

In the operating room, the previously planned stereotactic coordinates were transferred to the CRW simulator. The stereotactic surgical arc was assembled, and a single burr hole was made under stereotactic conditions. A microrecording system was then placed, and a recording microelectrode was introduced to identify neurophysiological signals consistent with GPi activity. The sensorimotor region of the GPi was localized using preoperative MRI-based stereotactic coordinates combined with intraoperative microelectrode recording (MER). One or two planned trajectories were used per patient, with additional passes performed as needed to delineate borders and identify characteristic GPi neuronal firing patterns. Somatosensory responses were evaluated to confirm motor areas, and macrostimulation was used to assess proximity to the optic tract and posterior limb of the internal capsule, as well as to determine thresholds for side effects. Borders of the GPi, external globus pallidus (GPe), and medullary lamina were localized based on these recordings.

Intraoperative macrostimulation was performed to evaluate motor responses and safety. The minimal current at which a motor response (rigidity reduction) was observed was typically 1.5 mA; stimulation was then gradually increased up to 4 mA to assess tolerability and detect potential adverse effects. This standardized stimulation range (1–4 mA) was applied consistently across patients.

**Fig. 1.**
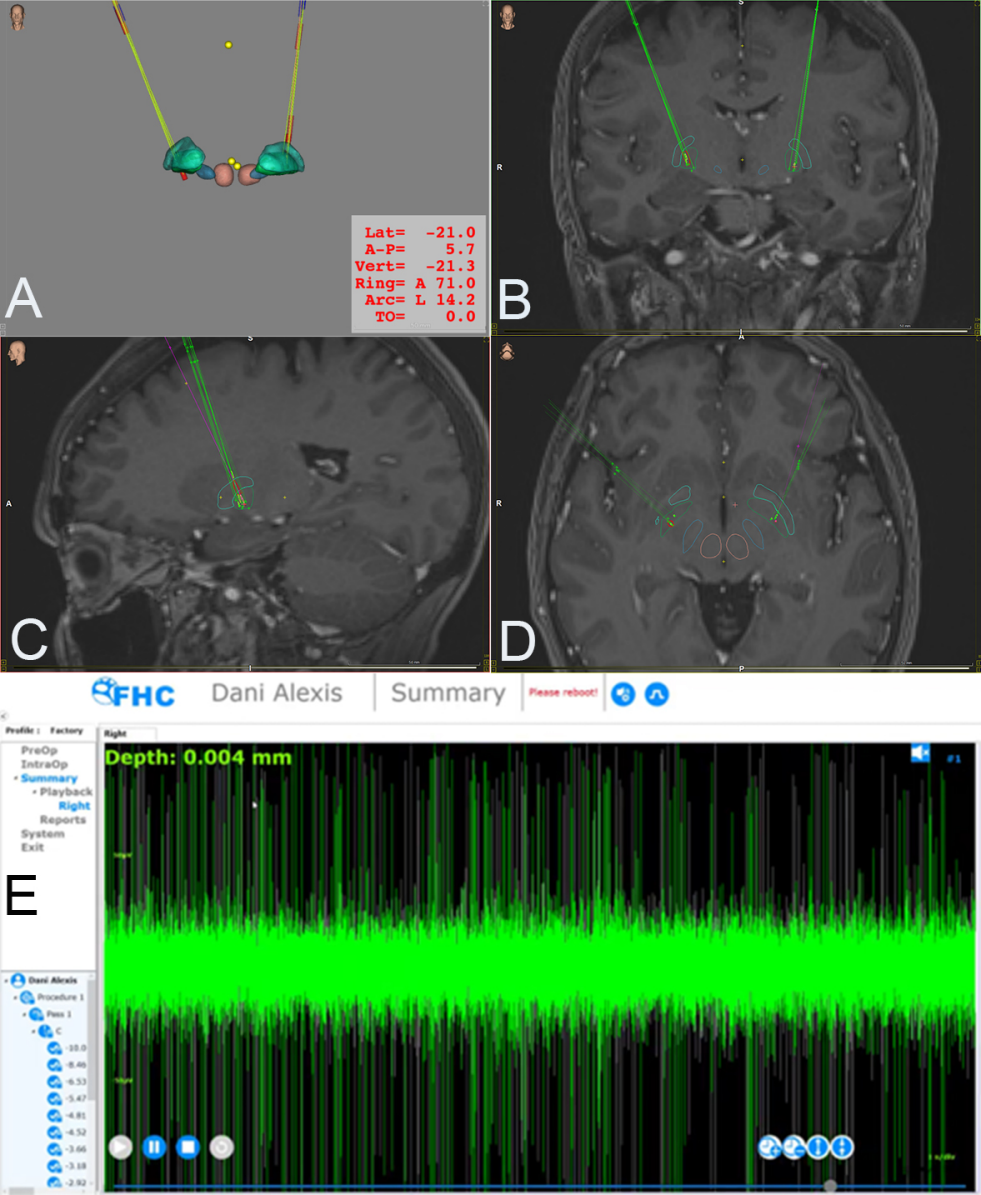
**Anatomical localization and neurophysiological confirmation of GPi ablations in patients with Parkinson’s disease**. Three-dimensional reconstructions (A) and multiplanar magnetic resonance image fusion views (B–D) illustrating the anatomical localization of unilateral pallidotomy lesions within the internal globus pallidus (GPi). Panel (E) shows intraoperative microelectrode recording, demonstrating the characteristic irregular high-frequency firing pattern of internal pallidal neurons at the target site. Lat: lateral coordinate; A–P: anteroposterior coordinate; Vert: vertical coordinate; Ring: ring angle of the stereotactic frame; Arc: arc angle of the stereotactic frame; TO: target offset (depth to the target).

Finally, two or three radiofrequency lesions were applied at 80 °C for 90 seconds at sites showing the best concordance between neurophysiological findings and intraoperative clinical responses. Ablations were performed with an Inomed radiofrequency lesion generator using a bipolar electrode (see Fig. 1).

## 3. Results

### 3.1 Demographic Data

The cohort included 12 patients with advanced Parkinson’s disease (7 males and 5 females) who underwent unilateral pallidotomy (6 right-sided and 6 left-sided). The mean age at surgery was 56.9 ± 9.8 years, and the average disease duration was 9.4 ± 3.8 years (see Table 1).

**Table 1.** **Demographic data, MDS-UPDRS-III scores in ON and OFF states, and NEUROPSI total scores before and after unilateral pallidotomy**.

Disease duration (years)	Side	Pre-OFF	Post-OFF	Δ OFF	% OFF	Pre-ON	Post-ON	Δ ON	% ON	NEUROPSI pre	NEUROPSI post	Δ NEUROPSI
8	Right	30	13	17	56.7	27	13	14	51.9	8.67	9.39	0.72
18	Right	48	30	18	37.5	14	6	8	57.1	5.33	5.89	0.56
10	Right	70	65	5	7.1	3	3	0	0.0	8.33	10.00	1.67
8	Right	57	32	25	43.9	18	9	9	50.0	9.61	8.56	–1.05
8	Right	76	52	24	31.6	48	18	30	62.5	8.44	8.57	0.13
6	Right	41	28	13	31.7	10	4	6	60.0	8.62	8.75	0.13
6	Left	97	67	30	30.9	24	21	3	12.5	8.48	8.65	0.17
14	Left	61	53	8	13.1	13	3	10	76.9	8.80	8.92	0.12
13	Left	34	20	14	41.2	22	6	16	72.7	9.06	7.61	–1.45
8	Left	71	34	37	52.1	29	15	14	48.3	8.33	7.44	–0.89
5	Left	43	9	34	79.1	21	17	4	19.0	8.50	8.00	–0.50
9	Left	18	8	10	55.6	7	2	5	71.4	9.44	9.39	–0.05

This table presents demographic and clinical data for 12 patients with advanced Parkinson’s disease who underwent unilateral pallidotomy. Substantial motor improvements were observed in both OFF- and ON-medication states, as reflected by significant reductions in MDS-UPDRS Part III scores. Neuropsychological performance, as measured by the total NEUROPSI score, remained globally stable, with only minor postoperative variations, suggesting preservation of cognitive functions after surgery.

### 3.2 Primary Outcomes

**Fig. 2.**
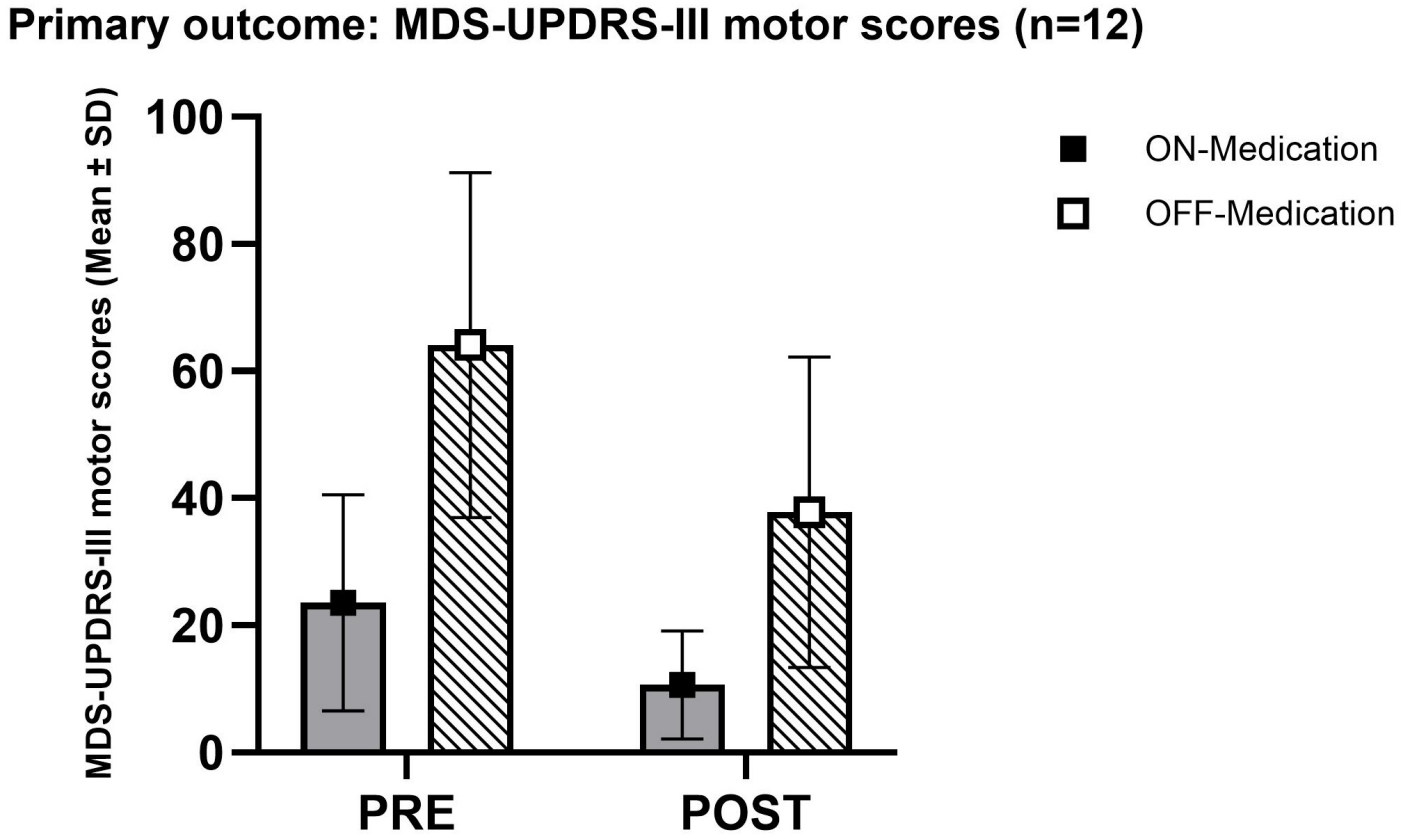
**Primary motor outcome after unilateral pallidotomy**. Mean MDS-UPDRS-III motor scores (± SD) in patients with advanced Parkinson’s disease, assessed in OFF- and ON-medication states before and after surgery. White bars with diagonal hatching represent OFF-medication scores, and gray bars represent ON-medication scores. Error bars indicate standard deviations. MDS-UPDRS-III, Unified Parkinson’s Disease Rating Scale Part III.

Mean MDS-UPDRS-III scores decreased from 64.1 ± 27.1 preoperatively (OFF medication) to 37.8 ± 24.4 postoperatively, and from 23.5 ± 17.0 to 10.6 ± 8.5 in the ON state. The global OFF-state improvement was 44.4% ± 21.2%. Paired *t*-test confirmed a highly significant reduction in motor scores (*t* = 6.19, *p* < 0.0001), with a mean change of –26.3 points (95% CI –34.7 to –18.0) (see Table 1 and Fig. 2).

### 3.3 Secondary Outcomes

In the OFF-medication state, all secondary motor outcomes showed significant improvement following unilateral pallidotomy. Contralateral rigidity decreased from 2.58 to 1.33 (mean change –1.25 points; 95% CI –1.83 to –0.67; *p* = 0.0006), representing a 48% reduction. Contralateral bradykinesia improved from 3.13 to 1.58 (mean change –1.54 points; 95% CI –2.04 to –1.04; *p* < 0.0001), corresponding to a 49.5% reduction. Resting tremor scores decreased from 1.13 to 0.21 (mean change –0.92 points; 95% CI –1.47 to –0.36; *p* = 0.004), an 81% improvement. Gait scores improved from 2.42 to 1.75 (mean change –0.67 points; 95% CI –1.16 to –0.17; *p* = 0.013), reflecting a 27.8% reduction. Postural stability also improved significantly, decreasing from 2.33 to 1.42 (mean change –0.91 points; 95% CI –1.64 to –0.18; *p* = 0.021), a 39% reduction.

**Fig. 3.**
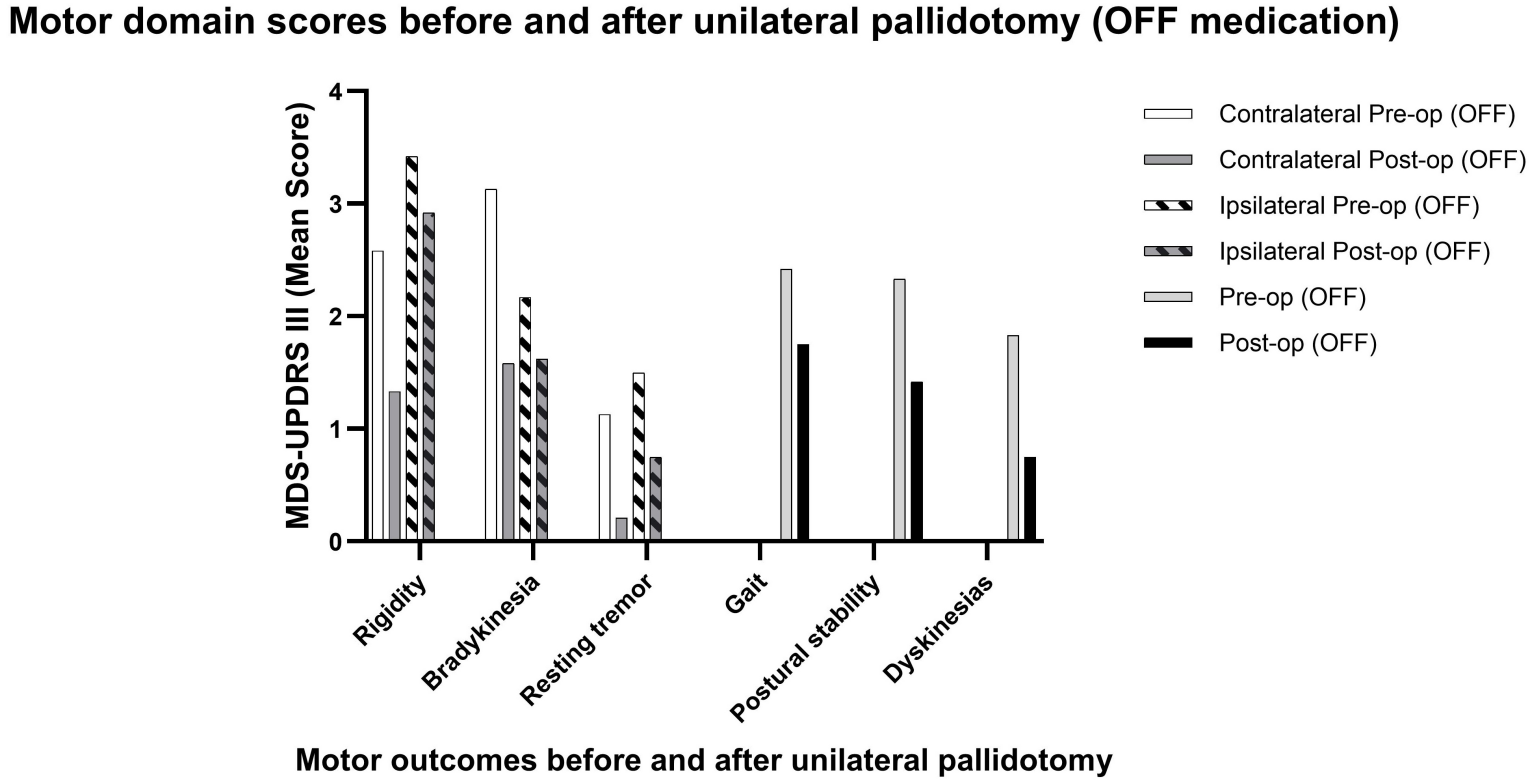
**Motor domain scores before and after unilateral pallidotomy (OFF medication)**.

In the ON-medication state, dyskinesia severity improved substantially, with mean scores decreasing from 1.83 to 0.75 (mean change –1.08 points; 95% CI –1.70 to –0.46; *p* = 0.017), representing an overall 56% reduction across the cohort (See Fig. 3 and Table 2).

**Table 2.** **Secondary motor outcomes contralateral to the lesion before and after unilateral pallidotomy**.

Domain	Preoperative (mean ± SD)	Postoperative (mean ± SD)	% Improvement	Mean change (95% CI)	*p* value
Rigidity (OFF)	2.58 ± 0.74	1.33 ± 0.65	48.00%	–1.25 (–1.83 to –0.67)	0.0006
Bradykinesia (OFF)	3.13 ± 0.87	1.58 ± 0.79	49.50%	–1.54 (–2.04 to –1.04)	<0.0001
Resting tremor (OFF)	1.13 ± 0.72	0.21 ± 0.41	81.00%	–0.92 (–1.47 to –0.36)	0.004
Gait (OFF)	2.42 ± 0.51	1.75 ± 0.62	27.80%	–0.67 (–1.16 to –0.17)	0.013
Postural stability (OFF)	2.33 ± 0.52	1.42 ± 0.52	39.00%	–0.91 (–1.64 to –0.18)	0.021
Dyskinesias (ON)	1.83 ± 0.75	0.75 ± 0.50	56.00%	–1.08 (–1.70 to –0.46)	0.017

CI, confidence intervals.

### 3.4 Ipsilateral Motor Outcomes

In addition to the primary contralateral motor outcomes, we also explored ipsilateral motor changes as secondary outcomes, in order to assess potential bilateral effects of unilateral pallidotomy.

In the pooled cohort (n = 12), there was a trend toward postoperative improvement in all ipsilateral motor domains, although none of the changes reached statistical significance. Mean rigidity scores decreased from 3.42 ± 1.98 to 2.92 ± 2.57 (14.6% improvement, *p* = 0.519). Ipsilateral bradykinesia, measured by the average of finger and toe tapping tasks, was reduced from 2.17 ± 1.15 to 1.62 ± 1.26 (25.0% improvement, *p* = 0.084). Rest tremor improved from 1.50 ± 1.68 to 0.75 ± 1.76 (50.0% reduction, *p* = 0.121) (See Fig. 3 and Table 3).

**Table 3.** **Ipsilateral motor outcomes in the OFF-medication state (n = 12)**.

Variable	Pre (mean ± SD)	Post (mean ± SD)	Mean diff	95% CI	*p*-value	Effect size (dz)	Test
Rigidity	3.42 ± 1.98	2.92 ± 2.57	–0.50	[–1.67, 0.83]	0.5186	–0.22	Wilcoxon
Bradykinesia	2.17 ± 1.15	1.62 ± 1.26	–0.54	[–1.10, 0.02]	0.0840	–0.55	paired *t*-test
Rest tremor	1.50 ± 1.68	0.75 ± 1.76	–0.75	[–1.62, 0.12]	0.1207	–0.49	paired *t*-test

When analyzed separately by surgical side, heterogeneous results were observed. In right-sided pallidotomy (n = 6), rigidity decreased from 3.83 ± 2.04 to 2.50 ± 1.97 (34.8% improvement, *p* = 0.219), bradykinesia improved modestly from 2.25 ± 1.04 to 1.83 ± 1.29 (18.5% improvement, *p* = 0.383), and rest tremor decreased from 1.83 ± 1.83 to 1.00 ± 2.45 (45.5% reduction, *p* = 0.363). In left-sided pallidotomy (n = 6), rigidity slightly worsened from 3.00 ± 2.00 to 3.33 ± 3.20 (–11.1%, *p* = 0.771), whereas bradykinesia showed a reduction from 2.08 ± 1.36 to 1.42 ± 1.32 (32.0% improvement, *p* = 0.109), and rest tremor decreased from 1.17 ± 1.60 to 0.50 ± 0.84 (57.1% reduction, *p* = 0.157).

Bars represent mean MDS-UPDRS scores in the OFF state for rigidity, bradykinesia, and rest tremor (contralateral and ipsilateral), as well as axial domains (gait, postural stability) and dyskinesias in the ON state. Contralateral symptoms improved markedly across all domains, with reductions of more than 50% in rigidity, bradykinesia, and tremor. Ipsilateral changes were less pronounced, but trends toward improvement were observed, particularly in bradykinesia and rest tremor. Axial domains also showed partial postoperative benefit, while time spent with dyskinesias was consistently reduced. Taken together, these findings suggest that unilateral pallidotomy not only provides robust contralateral motor relief, but may also exert modest ipsilateral and axial effects, possibly reflecting broader network-level modulation.

Values are presented as mean ± standard deviation. Outcomes include rigidity, bradykinesia, resting tremor, gait, and postural stability (all assessed in the OFF-medication state contralateral to the lesion), and dyskinesias (assessed in the ON-medication state). Percentage improvement, mean change with 95% confidence intervals, and *p* values from paired tests are shown. Significant improvements were observed across all domains, reflecting consistent contralateral motor benefit after pallidotomy.

Although unilateral pallidotomy is primarily intended to improve contralateral symptoms, we also explored potential ipsilateral effects. Mean rigidity scores decreased slightly (–0.50 points), bradykinesia showed a moderate reduction (–0.54 points, dz = –0.55) with a trend toward significance (*p* = 0.084), and rest tremor was reduced by half (–0.75 points, dz = –0.49), though without reaching statistical significance. These findings suggest that unilateral pallidotomy may exert a limited but consistent ipsilateral effect, particularly on bradykinesia and rest tremor, reinforcing the hypothesis of interhemispheric motor network modulation.

### 3.5 Cognitive Outcomes

**Fig. 4.**
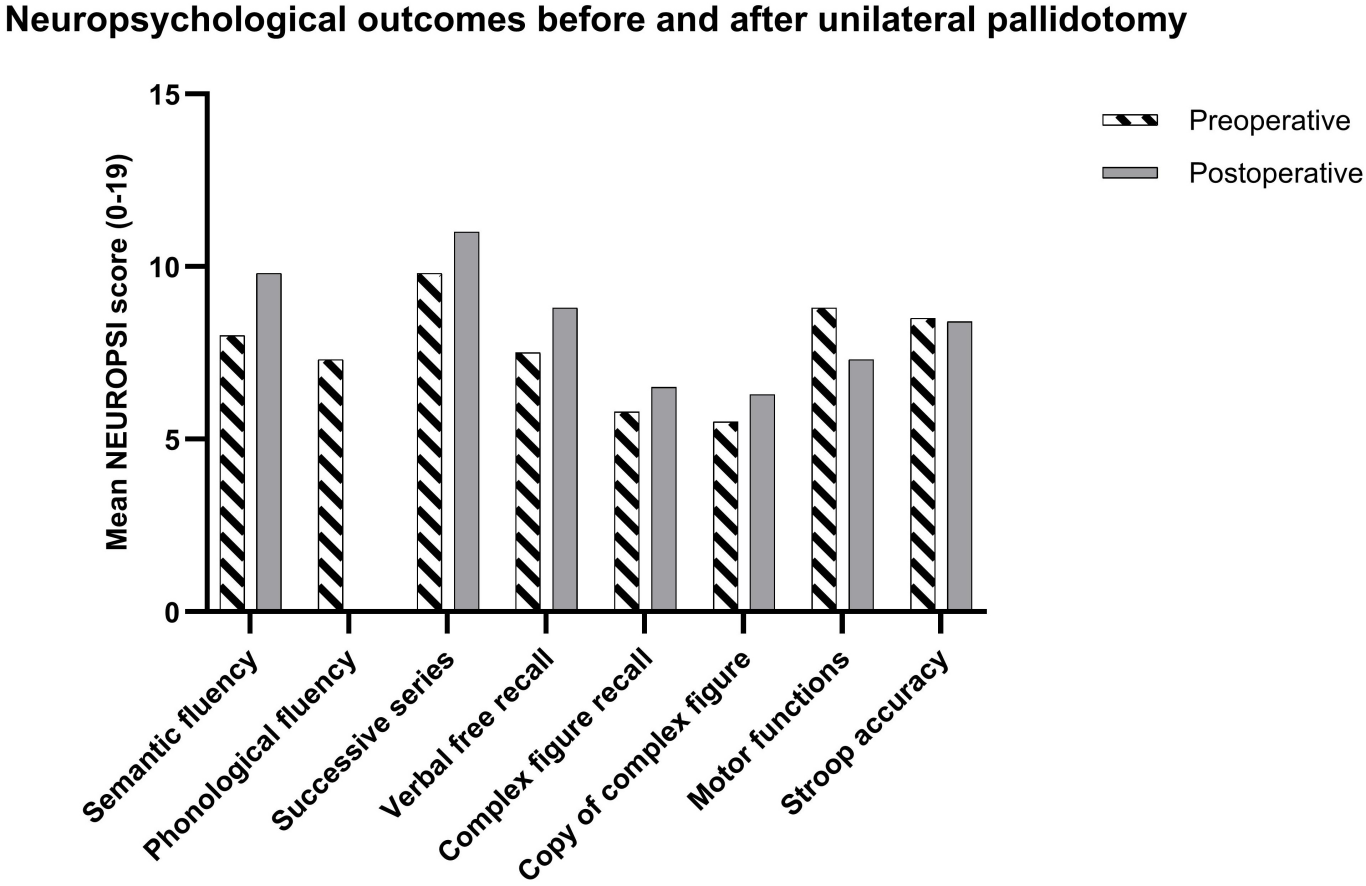
**Neuropsychological outcomes before and after unilateral pallidotomy**.

Across the cohort (n = 12), orientation in time, space, and person remained intact at ceiling levels pre- and postoperatively. The most consistent improvements were found in executive-verbal domains. Semantic fluency increased from 8.0 ± 3.1 to 9.8 ± 3.0 (Δ +1.8; 95% CI –1.2 to +4.7; *dz* = 0.84), and phonological fluency from 7.3 ± 2.8 to 8.8 ±2.6 (Δ +1.5; 95% CI –0.6 to +3.6; *dz* = 0.93). Successive series also improved (9.8 ± 2.7 to 11.0 ± 2.6; Δ +1.3; 95% CI –1.1 to +3.6; *dz* = 0.72). Memory outcomes were mixed: verbal free recall improved modestly (7.5 ± 2.9 to 8.8 ± 3.0), whereas recognition and the verbal learning curve remained stable. Visuospatial copying showed a small increase (5.5 ± 3.2 to 6.3 ± 3.4), while motor functions tended to decline. Stroop interference measures did not differ significantly (See Fig. 4 and Table 4).

**Table 4.** **Global neuropsychological outcomes before and after unilateral pallidotomy (n = 12)**.

Domain	Preoperative mean ± SD	Postoperative mean ± SD	Mean Δ (95% CI)	dz
Semantic fluency	8.0 ± 3.1	9.8 ± 3.0	+1.8 (−1.2, +4.7)	0.84
Phonological fluency	7.3 ± 2.8	8.8 ± 2.6	+1.5 (−0.6, +3.6)	0.93
Successive series	9.8 ± 2.7	11.0 ± 2.6	+1.3 (−1.1, +3.6)	0.72
Verbal free recall	7.5 ± 2.9	8.8 ± 3.0	+1.3 (−0.9, +3.4)	0.67
Complex figure recall	5.8 ± 2.8	6.5 ± 2.7	+0.8 (−4.5, +6.0)	0.21
Copy of complex figure	5.5 ± 3.2	6.3 ± 3.4	+0.8 (−2.4, +3.9)	0.20
Motor functions	8.8 ± 3.1	7.3 ± 3.6	−1.5 (−5.0, +2.0)	–0.40
Stroop interference (accuracy)	8.5 ± 2.7	8.4 ± 2.8	−0.1 (−3.2, +3.0)	–0.05

Mean NEUROPSI scores are shown for semantic fluency, phonological fluency, successive series, verbal free recall, complex figure recall, copy of complex figure, motor functions, and Stroop interference accuracy. White bars with diagonal hatching represent preoperative scores, and gray bars represent postoperative scores. Improvements were most evident in fluency and working memory domains, while motor performance showed a mild decline. At the time of neuropsychological assessment, patients were not required to be in the ON-medication state due to variability in interview and testing schedules.

## 4. Discussion

### 4.1 Medium- and Long-Term Motor Efficacy of Ablative Pallidotomy vs. DBS and HIFU

All three techniques—pallidotomy, deep brain stimulation (DBS), and high-intensity focused ultrasound (HIFU)—provide significant motor benefits in advanced Parkinson’s disease, although they differ in magnitude, durability, and side-effect profile.

Unilateral, microelectrode-guided posteroventral pallidotomy has consistently shown substantial contralateral motor improvements and dyskinesia control. Molinuevo *et al*. [8] reported ≈37% OFF-state MDS-UPDRS-III improvement at 3 months, decreasing to ≈27% at 1 year. Fine and colleagues likewise documented sustained reductions in rigidity, bradykinesia, and dyskinesias without loss of ON-period benefit [9]. Vitek *et al*. [10] observed a 32% motor improvement at 6 months following unilateral pallidotomy compared with ≈5% in patients receiving medical treatment.

In our series, the magnitude of contralateral improvement was somewhat higher than in these previous reports, both in the OFF and ON states, encompassing rigidity, bradykinesia, and tremor. Additionally, exploratory analyses revealed modest ipsilateral changes—most evident in bradykinesia and rest tremor—suggesting a possible contribution of interhemispheric or network-level modulation. Axial domains such as gait and postural stability also showed partial postoperative benefit, while dyskinesia time in the ON state was consistently reduced. These findings suggest that unilateral pallidotomy may extend beyond purely contralateral effects, although the ipsilateral and axial improvements were of smaller magnitude and did not reach statistical significance.

When compared to DBS targeting the GPi, our outcomes are of similar magnitude. Lachenmayer *et al*. [11] reported a 29.8% MDS-UPDRS-III reduction at 6–12 months, while the randomized trial by Odekerken *et al*. [12] showed mean OFF-state UPDRS improvements of 11.4 and 20.3 points for GPi-DBS and STN-DBS, respectively—both significantly superior to baseline. Long-term studies and the German S3 guideline (2023) confirm that GPi-DBS maintains efficacy in controlling dyskinesias and OFF time [13]. Likewise, GPi-targeted HIFU has shown promising results, with a recent meta-analysis demonstrating significant improvements in UPDRS-II, -III, and -IV scores [14]. Although long-term evidence remains limited, the magnitude of HIFU outcomes appears comparable to that of DBS and pallidotomy.

Taken together, these findings reinforce the view that ablative pallidotomy, DBS, and HIFU each achieve meaningful motor benefits in advanced Parkinson’s disease with rigidity-akinetic predominance. Our current results support the established efficacy of pallidotomy, highlight possible ancillary ipsilateral and axial effects, and underscore the need for further studies with larger samples to clarify the durability and full spectrum of benefits relative to other surgical modalities.

### 4.2 Neuropsychology

Neuropsychological outcomes after unilateral pallidotomy demonstrated a selective pattern: executive and fluency domains showed the clearest improvement, while most other cognitive functions remained stable. In contrast, motor performance scores showed a mild postoperative decline. This does not reflect a true worsening of motor function—given that all patients exhibited marked clinical motor improvement—but rather likely represents a testing-related effect, as tasks within the motor domain of the neuropsychological battery require fine motor speed and dexterity that may remain sensitive to postoperative microlesional effects, fatigue, or fluctuations in dopaminergic state at the moment of evaluation. Importantly, orientation and recognition memory were preserved, indicating that core attentional and memory-retrieval processes were not adversely affected.

These findings align with previous reports suggesting that focal pallidal lesions may enhance executive–verbal functioning through network-level modulation while leaving other domains largely unchanged. Interpretation, however, is constrained by the small sample size (n = 12) and the retrospective nature of the study. In particular, information on ON/OFF medication status at the time of each neuropsychological assessment was not consistently available, introducing an unavoidable source of bias in motor-dependent tasks. Future studies with larger cohorts and standardized cognitive evaluations under controlled medication states are needed to confirm these trends and better define their clinical significance.

Unilateral pallidotomy with microrecording has been associated with a low risk of cognitive impairment [15], a risk that increases with bilateral interventions, which are currently discouraged. Studies such as that by Santana [16] found no significant cognitive alterations using event-related potentials (P300), supporting the neuropsychological safety of the procedure. In the series by Strutt, patients who underwent unilateral pallidotomy continued to experience long-term motor benefits after surgery, while only mild neuropsychological declines were reported [17]. The cognitive effects of GPi-targeted HIFU remain poorly understood, but are presumed to carry a low risk, similar to that of radiofrequency pallidotomy [14].

In summary, unilateral pallidotomy demonstrates a safe cognitive profile and may be considered a valid therapeutic alternative, particularly in settings where other options such as DBS or HIFU are unavailable or inaccessible.

### 4.3 Accessibility and Relative Costs

Classic pallidotomy stands out for its low initial cost, as it does not require implanted hardware or periodic replacements. According to Spindola *et al*. [18], the cost per 1% improvement in UPDRS is nearly eight times lower with ablative pallidotomy than with DBS.

In Guatemala, at the Center for Epilepsy and Functional Neurosurgery, the cost of a unilateral ablative pallidotomy is approximately USD 20,000, including preoperative studies, hospitalization, surgery, and follow-up. In comparison, the estimated cost of DBS in Guatemala is approximately USD 40,000–45,000, reflecting the additional expense of the device and surgical procedure.

For context, reported costs from other countries include DBS in the United States (USD 40,942.85 ± USD 17,987.43) [19] and HIFU in Canada (CAD 14,831, USD 10,700–10,800) [20]. These figures are provided for general perspective, but direct comparisons across countries and currencies are not strictly valid.

Within this local context, pallidotomy represents a more accessible surgical option, particularly in resource-limited settings, while DBS remains less available due to higher costs.

### 4.4 Safety Profile and Complications

DBS is associated with a low rate of major complications. In the series by Kenney *et al*. [21], intracranial hemorrhage was reported in 0.6% of cases and system-related infections in 4.4%. Long-term complications related to DBS device implantation that did not require additional surgery included hardware discomfort (1.1%) and loss of therapeutic effect (1.4%). Hardware-related complications requiring surgical revision included wound infections (1.7%), electrode misplacement and/or migration (1.7%), component fracture (1.4%), component malfunction (0.5%), and loss of effect (2.6%) [22].

HIFU eliminates the risk of hemorrhage associated with intracranial penetration. In the series by Thomas *et al*. [23], the most commonly observed adverse events were dysarthria or dysphagia (7.4%), gait disturbances (3.2%), weakness (3.2%), and taste disturbances (2.1%).

Microelectrode-guided radiofrequency pallidotomy, by avoiding implanted hardware, eliminates the risks of device malfunction or fibrosis. In our series, no major surgical complications such as hemorrhage or infection were observed. Because pallidotomy is an irreversible procedure, patients were systematically monitored intraoperatively for potential complications, including sensory disturbances, language impairment, increased rigidity, short-term memory alterations, and visual disturbances. Immediate postoperative CT scans were also obtained to exclude hemorrhage or other structural complications, and no significant adverse events were detected.

Lesion-based side effects may be irreversible, unlike hardware- or stimulation-related effects observed with DBS, which are often reversible. The use of microelectrode recording (MER) carries inherent procedural risks, which were carefully managed. Comparisons with previously published DBS and HIFU cohorts are presented for context, emphasizing relative safety without overstating outcomes in this series.

## 5. Conclusion

Our experience, in line with the studies by Llumiguano *et al*. [24], Huang *et al*. [25], and Eskandar *et al*. [26], confirms that unilateral microelectrode-guided pallidotomy, using imaging techniques (magnetic resonance imaging and computed tomography) and intraoperative neurophysiological monitoring through microrecording, is a safe and effective technique for the treatment of advanced Parkinson’s disease. The clinical outcomes obtained are comparable to those reported with deep brain stimulation (DBS), as noted by Krauss and Wolff Fernandes [27].

Despite these findings, we observed that with the resurgence of pallidotomy, preference has often been given to other, more costly and less accessible ablative techniques such as radiosurgery and high-intensity focused ultrasound (HIFU). Although these methods offer advantages such as non-invasiveness, they present limitations in terms of availability and cost, particularly in resource-limited settings.

In this context, it is pertinent to question why these options are preferred over pallidotomy, which has proven to be an effective and more accessible alternative. It is essential to consider factors such as resource availability, the experience of the medical team, and the individual characteristics of each patient when selecting the most appropriate therapeutic option.

It is important to emphasize that unilateral microelectrode-guided pallidotomy remains a valid and effective therapeutic option for patients with advanced Parkinson’s disease, especially in settings where more expensive techniques are not available. Its efficacy and safety position it as an alternative that deserves to be reconsidered in current clinical practice.

Our retrospective observational experience, consistent with previous studies [24, 25, 26], demonstrates that unilateral microelectrode-guided pallidotomy, using imaging guidance (MRI and CT) and intraoperative neurophysiological monitoring, provides clinically meaningful improvements in contralateral motor function and selected neuropsychological domains in patients with advanced Parkinson’s disease. While other ablative techniques such as radiosurgery or high-intensity focused ultrasound (HIFU) offer certain advantages, their availability and cost may limit accessibility in resource-constrained settings. Our findings support the continued use of pallidotomy as a viable therapeutic option, particularly in contexts where no specialized personnel are available to perform deep brain stimulation (DBS) programming protocols, where internet access for remote follow-up is not feasible, where symptoms are predominantly unilateral, and where alternative procedures cannot be implemented. These results highlight that optimal outcomes depend on careful patient selection and management by an experienced multidisciplinary team. Overall, they reinforce previously reported efficacy and safety profiles but do not establish new benchmarks or imply superiority over other surgical modalities.

## 6. Limitations

As this was a retrospective study, no prospective power calculation was conducted. Instead, we provide observed effect sizes and confidence intervals to contextualize the precision of our findings. The small cohort size (n = 12) increases the likelihood of inflated effect sizes and precludes definitive conclusions. The observed improvements should therefore be considered preliminary, emphasizing the need for case accumulation and longer follow-up. With a larger dataset, more robust analyses stratified by ON/OFF medication state and surgical laterality will be possible to better define the true therapeutic profile and durability of pallidotomy.

## Availability of Data and Materials

The datasets generated during the present study are not publicly available due to ethical restrictions and patient confidentiality. Access to the data may be granted by the corresponding author upon reasonable request and in accordance with institutional ethical guidelines.

## References

[1] Simon DK, Tanner CM, Brundin P (2020). Parkinson Disease Epidemiology, Pathology, Genetics, and Pathophysiology. *Clinics in Geriatric Medicine*.

[2] Bakay RAE, Bakay RAE (2009). History of surgery for movement disorders. *Movement Disorder Surgery: The Essentials*.

[3] Cooper IS (1953). Ligation of the anterior choroidal artery for involuntary movements; parkinsonism. *The Psychiatric Quarterly*.

[4] Spiegel EA, Wycis HT, Marks M, Lee AJ (1947). Stereotaxic Apparatus for Operations on the Human Brain. *Science (New York, N.Y.)*.

[5] Narabayashi H, Okuma T (1953). Procaine oil blocking of the globus pallidus for the treatment of rigidity and tremor of parkinsonism. *Proceedings of the Japan Academy*.

[6] Guiot G, Brion S (1953). Treatment of abnormal movement by pallidal coagulation. *Revue Neurologique*.

[7] Svennilson E, Torvik A, Lowe R, Leksell L (1960). Treatment of parkinsonism by stereotatic thermolesions in the pallidal region. A clinical evaluation of 81 cases. *Acta Psychiatrica Scandinavica*.

[8] Molinuevo JL, Valldeoriola F, Rumià J, Nobbe FA, Ferrer E, Tolosa E (2000). Efficacy and safety of posteroventral pallidotomy for the treatment of advanced Parkinson’s disease. *Medicina Clinica*.

[9] Fine J, Duff J, Chen R, Chir B, Hutchison W, Lozano AM (2000). Long-term follow-up of unilateral pallidotomy in advanced Parkinson’s disease. *The New England Journal of Medicine*.

[10] Vitek JL, Bakay RAE, Freeman A, Evatt M, Green J, McDonald W (2003). Randomized trial of pallidotomy versus medical therapy for Parkinson’s disease. *Annals of Neurology*.

[11] Lachenmayer ML, Mürset M, Antih N, Debove I, Muellner J, Bompart M (2021). Subthalamic and pallidal deep brain stimulation for Parkinson’s disease-meta-analysis of outcomes. *NPJ Parkinson’s Disease*.

[12] Odekerken VJJ, van Laar T, Staal MJ, Mosch A, Hoffmann CFE, Nijssen PCG (2013). Subthalamic nucleus versus globus pallidus bilateral deep brain stimulation for advanced Parkinson’s disease (NSTAPS study): a randomised controlled trial. *The Lancet. Neurology*.

[13] Reese R, Koeglsperger T, Schrader C, Tönges L, Deuschl G, Kühn AA (2025). Invasive therapies for Parkinson’s disease: an adapted excerpt from the guidelines of the German Society of Neurology. *Journal of Neurology*.

[14] Abbas A, Hassan MA, Shaheen RS, Hussein A, Moawad MHED, Meshref M (2024). Safety and efficacy of unilateral focused ultrasound pallidotomy on motor complications in Parkinson’s disease (PD): a systematic review and meta-analysis. *Neurological Sciences*.

[15] Gironell A, Kulisevsky J, Rami L, Fortuny N, García-Sánchez C, Pascual-Sedano B (2003). Effects of pallidotomy and bilateral subthalamic stimulation on cognitive function in Parkinson disease. A controlled comparative study. *Journal of Neurology*.

[16] Santana D, Sandoval L, González G, Haro R, Ramírez Y, Jiménez Ponce F (2013). Effect of posteroventral pallidotomy on event-related P300 in Parkinson’s disease. *Gaceta Medica De Mexico*.

[17] Strutt AM, Lai EC, Jankovic J, Atassi F, Soety EM, Levin HS (2009). Five-year follow-up of unilateral posteroventral pallidotomy in Parkinson’s disease. *Surgical Neurology*.

[18] Spindola B, Leite MA, Orsini M, Fonoff E, Landeiro JA, Pessoa BL (2017). Ablative surgery for Parkinson’s disease: Is there still a role for pallidotomy in the deep brain stimulation era?. *Clinical Neurology and Neurosurgery*.

[19] Bishay AE, Lyons AT, Koester SW, Paulo DL, Liles C, Dambrino RJ (2024). Global Economic Evaluation of the Reported Costs of Deep Brain Stimulation. *Stereotactic and Functional Neurosurgery*.

[20] Meng Y, Pople CB, Kalia SK, Kalia LV, Davidson B, Bigioni L (2020). Cost-effectiveness analysis of MR-guided focused ultrasound thalamotomy for tremor-dominant Parkinson’s disease. *Journal of Neurosurgery*.

[21] Kenney C, Simpson R, Hunter C, Ondo W, Almaguer M, Davidson A (2007). Short-term and long-term safety of deep brain stimulation in the treatment of movement disorders. *Journal of Neurosurgery*.

[22] Fenoy AJ, Simpson RK (2014). Risks of common complications in deep brain stimulation surgery: management and avoidance. *Journal of Neurosurgery*.

[23] Thomas B, Bellini G, Lee WY, Shi Y, Mogilner A, Pourfar MH (2025). High Intensity Focused Ultrasound - Longitudinal Data on Efficacy and Safety. *Tremor and other Hyperkinetic Movements (New York, N.Y.)*.

[24] Llumiguano C, Dóczi T, Baths I (2006). “Microelectrode-guided stereotactic pallidotomy and pallido-thalamotomy for the treatment of Parkinson’s disease”. *Neurocirugia (Asturias, Spain)*.

[25] Huang YA, Yin Z, Zhang BG, Cheng GG, Wu C, Ma HW (2003). Stereotactic microelectrode-guided posteroventral pallidotomy for Parkinson’s disease. *Zhonghua Wai Ke Za Zhi*.

[26] Eskandar EN, Shinobu LA, Penney JB, Cosgrove GR, Counihan TJ (2000). Stereotactic pallidotomy performed without using microelectrode guidance in patients with Parkinson’s disease: surgical technique and 2-year results. *Journal of Neurosurgery*.

[27] Krauss JK, Wolff Fernandes F (2021). Svennilson’s Publication on Pallidotomy for Parkinsonism in 1960: A Most Influential Paper in the Field. *Movement Disorders Clinical Practice*.

